# Myo1e overexpression in lung adenocarcinoma is associated with increased risk of mortality

**DOI:** 10.1038/s41598-023-30765-y

**Published:** 2023-03-13

**Authors:** Ignacio Jusue-Torres, Richies Tiv, Julio C. Ricarte-Filho, Apurva Mallisetty, Leglys Contreras-Vargas, Maria Jose Godoy-Calderon, Karam Khaddour, Kathleen Kennedy, Klara Valyi-Nagy, Odile David, Martha Menchaca, Anastasia Kottorou, Angelos Koutras, Foteinos Dimitrakopoulos, Khaled M. Abdelhady, Malek Massad, Israel Rubinstein, Lawrence Feldman, John Stewart, Takeshi Shimamura, Ludmila Danilova, Alicia Hulbert

**Affiliations:** 1 Department of Neurological Surgery, Mayo Clinic, Rochester, MN USA; 2 Department of Surgery, University of Illinois at Chicago College of Medicine, Chicago, IL USA; 3 Cancer Center, University of Illinois at Chicago, Chicago, IL USA; 4 Division of Hematology Oncology, University of Illinois at Chicago College of Medicine, Chicago, IL USA; 5 Department of Pathology, University of Illinois at Chicago College of Medicine, Chicago, IL USA; 6 Department of Radiology, University of Illinois at Chicago College of Medicine, Chicago, IL USA; 7 Molecular Oncology Laboratory, Division of Oncology, Medical School, University of Patras, Patras, Greece; 8 Division of Cardiothoracic Surgery, University of Illinois at Chicago College of Medicine, Chicago, IL USA; 9 Medical and Research Services, Jesse Brown VA Medical Center, Chicago, IL USA; 10 Division of Pulmonary, Critical Care, Sleep, and Allergy Medicine, University of Illinois at Chicago College of Medicine, Chicago, IL USA; 11 Section of Surgical Oncology, Department of Surgery, Louisiana State University, New Orleans, LA USA; 12 Department of Oncology, The Sidney Kimmel Comprehensive Cancer Center, Johns Hopkins University School of Medicine, Baltimore, MD USA; 13 Laboratory of Systems Biology and Computational Genetics, Vavilov Institute of General Genetics, Russian Academy of Sciences, Moscow, Russia; 14 Department of Surgery, University of Illinois College of Medicine, 909 South Wolcott Ave, COMRB Suite 5140, Chicago, IL 60612 USA

**Keywords:** DNA methylation, Cancer epigenetics, Non-small-cell lung cancer, Prognostic markers, Tumour biomarkers

## Abstract

This study aims to perform a comprehensive genomic analysis to assess the influence of overexpression of *MYO1E* in non-small cell lung carcinoma (NSCLC) and whether there are differences in survival and mortality risk in NSCLC patients depending on both DNA methylation and RNA expression of MYO1E. The DNA methylation probe cg13887966 was inversely correlated with *MYO1E* RNA expression in both LUAD and LUSC subpopulations showing that lower *MYO1E* RNA expression was associated with higher MYO1E DNA methylation. Late stages of lung cancer showed significantly lower MYO1E DNA methylation and significantly higher *MYO1E* RNA expression for LUAD but not for LUSC. Low DNA methylation as well as high RNA expression of MYO1E are associated with a shorter median survival time and an increased risk of mortality for LUAD, but not for LUSC. This study suggests that changes in MYO1E methylation and expression in LUAD patients may have an essential role in lung cancer’s pathogenesis. It shows the utility of MYO1E DNA methylation and RNA expression in predicting survival for LUAD patients. Also, given the low normal expression of MYO1E in blood cells MYO1E DNA methylation has the potential to be used as circulating tumor marker in liquid biopsies.

## Introduction

Lung cancer is the second most common malignancy in the United States with an estimated annual incidence of 235,760 new cases in 2021 in both males and females. Death from lung cancer remains the leading cause of cancer-related death representing 22% of all cancer-related deaths. There has been a decrease in incidence and mortality in lung cancer for the last few years due to decreased smoking, the introduction of lung cancer low dose CT screening and the recent advances in targeted molecular therapies^1–3^. However, only 30% of non-small cell lung cancer (NSCLC) tissue specimens harbor EGFR mutations, while only 5% of patients with NSCLC have ALK gene rearrangements^4–8^. Therefore, most lung cancer patients will not benefit from these recent precision medicine advances which are based on specific DNA mutations^9^. Thus, there is an unmet need for a deeper understanding of the mechanisms implicated in NSCLC pathogenesis. Epigenetic changes in NSCLC have emerged as an important mechanism contributing to cancer initiation, proliferation, and invasiveness by modulating gene expression^10–12^. Myosin 1E (MYO1E) is a non-muscle class I myosin and actin-dependent molecular motor which binds to the cellular plasma membrane and serves as a scaffold for membrane protrusion^13^. MYO1E contributes to remodeling of the cellular membrane during endocytosis and exocytosis prompting cell migration and invasion^14–16^. Different studies have explored the importance of myosins and their potential role in cancer, regulating tumor formation, cell invasion, migration and metastasis^17^. Non-muscle myosins have been documented to be expressed in drug-resistant melanoma cell clones and their expression was associated with cell survival regardless of the therapy^18,19^. Specifically, MYO1E has oncogenic features, and its expression has been shown to be correlated with poor prognosis in patients with invasive breast cancer^20,21^. Despite this association with aggressiveness, the role of *MYO1E* expression in NSCLC has not been previously explored. Therefore, this study aims to perform a comprehensive genomic analysis to assess the influence of overexpression of *MYO1E* in NSCLC and whether there are differences in survival and mortality risk in NSCLC depending on both DNA methylation and RNA expression of *MYO1E*.

## Results

### Patients’ characteristics

A total of 1017 patients with NSCLC were identified in the TCGA database, 515 with LUAD and 502 with LUSC. Most patients were male (60%), Caucasian (73%) with median age 67 years (60–73 years) and median pack*years smoked 40 (29–60) (Table 1). These patients’ characteristics are similar to previous population-based epidemiological studies^22^. *MYO1E* RNA expression groups only showed differences in stages, showing that the low *MYO1E* RNA expression group had a higher percentage of patients with NSCLC stage I group and the high *MYO1E* RNA expression group, a higher proportion of patients with NSCLC stage II (p = 0.036). All the clinical characteristics in the validation cohort were comparable to the TCGA data (supplemental Table 1). Notably, African Americans were underrepresented (8%) in both cohorts.

**Table 1:** Baseline characteristics TCGA vs validation cohort.

Patient characteristics	Validation cohort (N = 127)	TCGA (N = 1017)
Age at diagnosis (years) (IQR)	66 (59–70)	67 (60–73)
Gender		
Male (%)	97 (76%)	609 (60%)
Female (%)	30 (34%)	408 (40%)
Race		
Caucasians (%)	90 (71%)	738 (73%)
African Americans (%)	35 (8%)	82 (8%)
Asians (%)	2 (2%)	17 (2%)
American Indian or Alaska native (%)	0 (0%)	1 (< 1%)
Non reported (%)	0 (0%)	179 (18%)
Pack-year (IQR)	40 (0–55)	40 (29–60)
Histology		
Adenocarcinoma (LUAD) (%)	66 (52%)	515 (51%)
Squamous-cell (LUSC) (%)	55 (48%)	502 (49%)
Stage		
I (%)	63 (50%)	520 (52%)
II (%)	31 (24%)	284 (28%)
III (%)	30 (24%)	168 (17%)
IV (%)	3 (2%)	33 (3%)

*IQR* interquartile range.

### Correlation of MYO1E DNA methylation and *MYO1E* RNA expression

**Figure 1:**
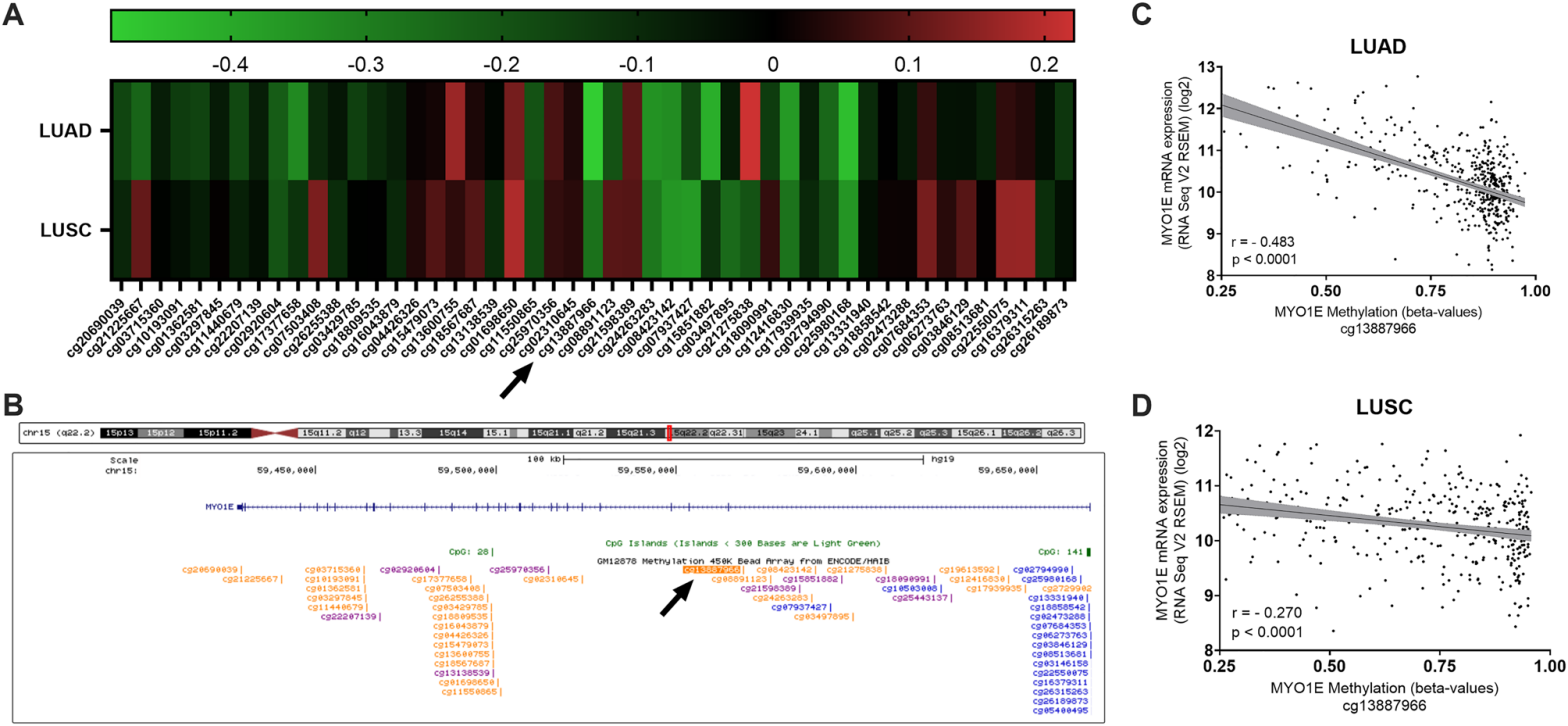
Strong inverse correlations are observed between cg13887966 DNA methylation intronic probe and *MYO1E* RNA expression for LUAD and LUSC. (**A**) Heatmap showing Spearman’s correlation coefficients for *MYO1E* RNA expression and all CpG dinucleotides probes for MYO1E DNA methylation ordered by genomic position in LUAD and LUSC. Red represents direct correlation and green inverse correlation (p < 0.05 “*”; p < 0.01 “**”; p < 0.001 “***”). (**B**) Schematic representation of the relative genomic position of the different CpG probes for MYO1E showing that cg13887966 is located on MYO1E’s 1st intron. (**C**) and (**D**) Scatterplots showing the correlation between RNA expression and cg13887966 probe for MYO1E DNA methylation in LUAD (**C**) and LUSC (**D**).

The *MYO1E* gene has 53 CpG probes on the Infinium HumanMethylation450 array. We correlated these probes with RNA expression of *MYO1E* in TCGA, and the CpG probe cg13887966 showed the strongest correlation in LUAD (Spearman’s r = −0.483, p-value < 0.0001) and ranked 5th in LUSC (Spearman’s r = −0.270, p-value < 0.001) (Fig. 1). CpG probe cg13887966 is located on *MYO1E*’s first intron (position: chr15:59568712) (Fig. 1). The DNA methylation probe cg13887966 was inversely correlated with *MYO1E* RNA expression in both LUAD and LUSC (Fig. 1) showing that lower *MYO1E* RNA expression is associated with high MYO1E DNA methylation. We used the median methylation level of this probe to split samples into high and low MYO1E DNA methylation groups for our further analysis.

Data derived from previous published wide genomic and transcriptomic human cell line studies shows that cell lines after being treated with 5-AZA significantly decrease MYO1E DNA methylation on probe cg13887966 (p = 0.0004) (GEO accession GSE202559^23^) and significantly increase *MYO1E* RNA expression (p = 0.0059) (GEO accession GSE62955 and GSE62958^24^) (Supplemental Fig. 1).

### Differential expression analysis

**Figure 2:**
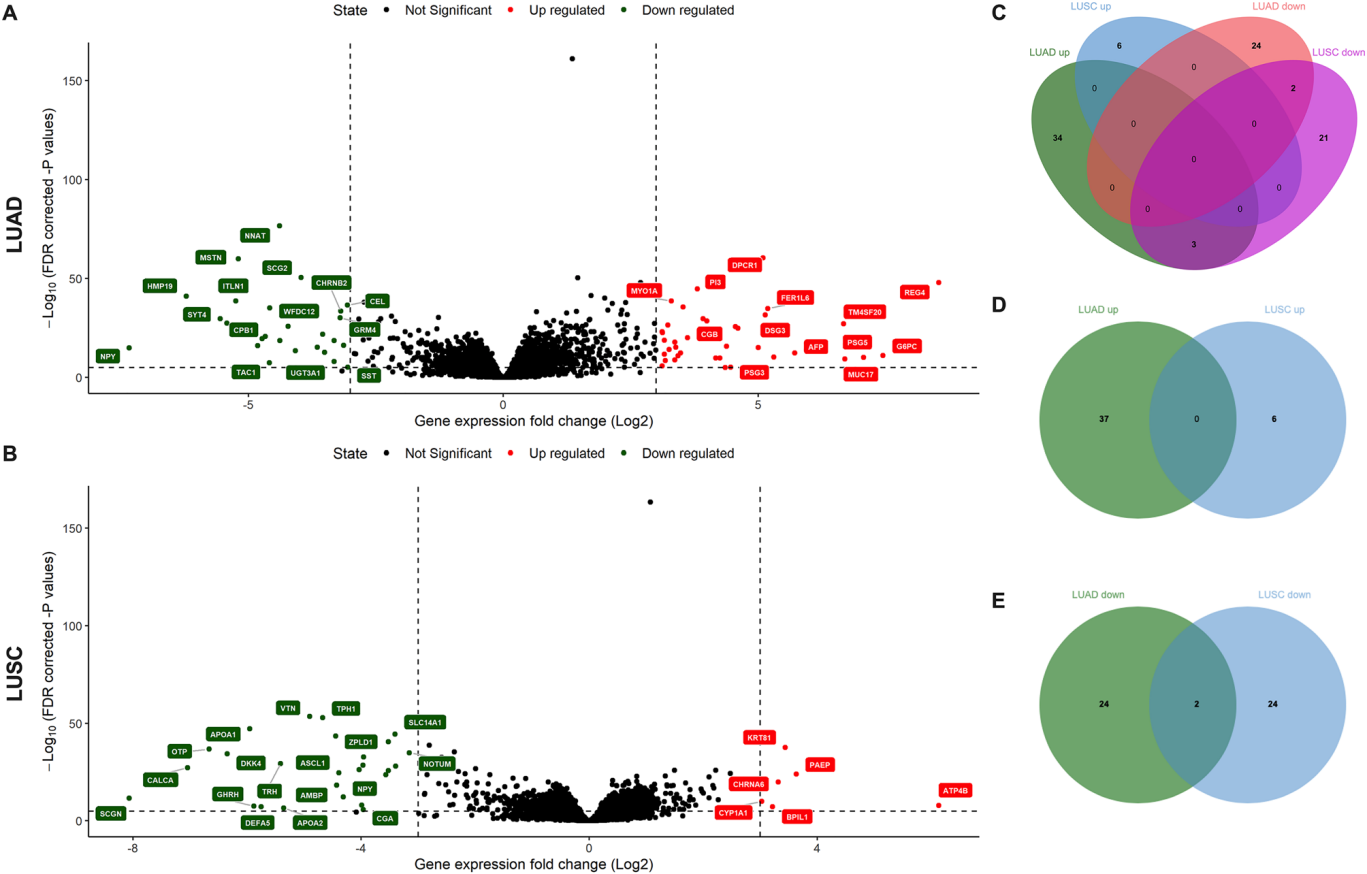
Volcano plots and Venn diagrams for differential expression analysis showing up- and down-regulated genes when *MYO1E* is overexpressed for LUAD (**A**) and LUSC (**B**) (FDR cutoff of 10^-5 and logFC of 3). Venn diagrams showing the overlap of genes between LUAD and LUSC for all genes (**C**), for upregulated genes (**D**) and downregulated genes (**E**).

Using the TCGA RNA seq data from LUAD and LUSC (Illumina HiSeq, RSEM normalized counts), we performed differential expression analysis of *MYO1E* expression high vs low in LUAD and LUSC (Fig. 2). When *MYO1E* was highly expressed, there were 26 down-regulated and 37 upregulated genes in LUAD, and there were 26 down-regulated and 6 upregulated genes in LUSC (Fig. 2, Supplemental Table 2). Among the upregulated genes, there was no overlap between LUAD and LUSC. Among the downregulated genes, there were two genes in common between LUAD and LUSC (NPY and CHGA).

### Correlation expression analysis

Additionally, using the TCGA RNA seq data, we assessed the correlation of expression between *MYO1E* and some of the most relevant oncogenic pathway genes in lung cancer including: *MAP2K1, MAPK1, MAPK3, KRAS, mTOR, AKT1, AKT2, RPS6, CDKN2A*, *RB1, p53, MYC, STK11, EGFR and KEAP1*^25,26^. The correlation analysis showed that *MYO1E* RNA expression was significantly positively correlated with the expression of *MAP2K1, RB1, KRAS, MYC, MTOR, EGFR* and *MAPK1* and significantly negatively correlated with *RPS6*, *TP53* and *CDKN2A* in LUAD (Supplemental Fig. 2). In LUSC, *MYO1E* RNA expression was significantly positively correlated with the expression of *MAP2K1, MTOR EGFR* and *MAPK1* and significantly negatively correlated with *RPS6*, *KEAP1* and *KRAS* (Supplemental Fig. 2). These results suggest that *MYO1E* RNA expression is associated with increased gene expression from oncogenic MAPK/ERK, MTOR and EGFR pathways and with decreased gene expression of RPS6 pathway in both LUAD and LUSC.

### RNA expression by cancer stage

**Figure 3:**
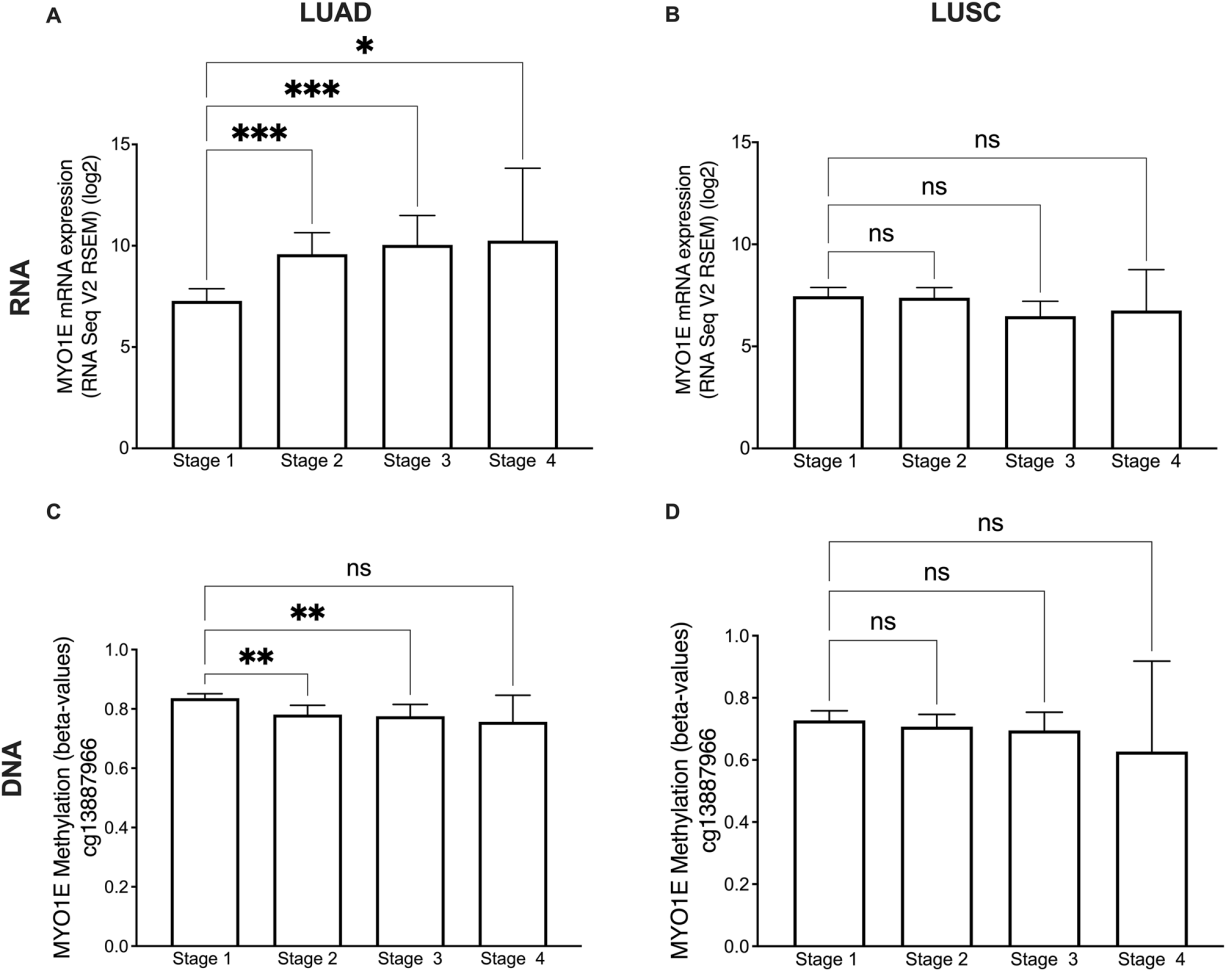
*MYO1E* RNA expression is significantly higher on late lung cancer stages in LUAD but not in LUSC and MYO1E DNA methylation is significantly lower on late lung cancer stages in LUAD but not in LUSC. Bar plots showing *MYO1E* RNA expression by stage for LUAD (**A**) and LUSC (**B**) and MYO1E DNA methylation by stage for LUAD (**C**) and LUSC (**D**) representing mean with 95% confidence interval. Multiple comparison ANOVA test. *NS* non-significant; *p < 0.05; **p < 0.01; ***p < 0.001.

Using the TCGA RNA seq data and DNA methylation data for the cg13887966 probe from LUAD and LUSC, we assessed the *MYO1E* RNA expression and the MYO1E DNA methylation by cancer stage. This analysis showed that *MYO1E* expression is significantly higher in late stages of LUAD, but not in LUSC, and that MYO1E DNA methylation is significantly lower in late stages of LUAD, but not in LUSC (Fig. 3).

### Normal *MYO1E* RNA expression

To examine the expression of *MYO1E* in various normal primary tissue types, we analyzed expression data of samples derived from non-disease tissues obtained from 838 donors using the Genotype-Tissue Expression (GTEx) project^27–29^. The lowest expression of *MYO1E* was observed in whole blood, brain and heart tissues in increasing order of magnitude, while arteries, skin and nerves exhibited the highest *MYO1E* expression levels. Normal lung tissue was the 20th highest expressing *MYO1E* (out of 53 tissues) (Supplemental Fig. 2). This observation indicates the potential of MYO1E to be used as circulating tumor marker in liquid biopsies given the low normal expression of *MYO1E* in blood cells.

### Survival analysis for RNA expression (caBIG, GEO and TCGA)

To determine how *MYO1E* expression correlates with overall survival, we used Kaplan–Meier Plotter (www.kmplot.com/lung), an online tool developed by Gyorffy et al.^30^. This tool performs survival meta-analysis of NSCLC from the cancer Biomedical Informatics Grid (caBIG), Gene Expression Omnibus (GEO) and The Cancer Genome Atlas (TCGA) repositories combined (n = 1925). There were significant differences in median overall survival (OS) in NSCLC when comparing *MYO1E* RNA expression low vs high. The log rank analysis showed statistically significant differences (p < 0.0001) with a median OS of 57 and 136 months for *MYO1E* RNA expression high and low, respectively (Supplemental Fig. 4). Univariate Cox proportional hazard analysis showed a higher mortality risk for NSCLC patients harboring higher *MYO1E* RNA expression compared with lower *MYO1E* RNA expression (HR 1.74, 95% CI: 1.48–2.05). The above findings were reproduced on multivariate Cox proportional hazard weighted analysis adjusted by histology, stage, sex, smoking history, and *MYO1E* RNA expression in NSCLC. As in univariate analysis, high *MYO1E* RNA overexpression in NSCLC was also associated with higher mortality risk when compared with those of lower *MYO1E* RNA expression (HR 1.69, 95% CI: 1.02–2.79) (Supplemental Fig. 4).

When comparing differences in *MYO1E* RNA expression in LUAD only (n = 719) in Kaplan–Meier Plotter, log-rank analysis showed statistically significant differences (p < 0.0001) with a median survival of 21 and 112 months for high vs low *MYO1E* RNA expression in LUAD, respectively (Supplemental Fig. 4). Univariate Cox proportional hazard analysis showed that higher mortality risk was observed for LUAD patients harboring high *MYO1E* RNA expression compared with low *MYO1E* RNA expression (HR 3.35, 95% CI: 2.44–4.6). The above findings were reproduced on multivariate Cox proportional hazard weighted analysis adjusted by stage, sex, smoking history, and MYO1E RNA expression in LUAD. High *MYO1E* RNA expression in LUAD adjusted by stage, sex and smoking history is associated with higher mortality risk when compared with low *MYO1E* RNA expression (HR 3.24, 95% CI: 2.15–4.89) (Supplemental Fig. 5).

When comparing differences in *MYO1E* RNA expression in LUSC only (n = 524) in Kaplan–Meier Plotter, log rank analysis showed no statistically significant differences (p = 0.48) with a median survival of 52 and 62 months for high vs low *MYO1E* RNA expression in LUSC, respectively (Supplemental Fig. 4). Univariate Cox proportional hazard analysis also showed no association with mortality risk for LUSC (HR 1.1, 95% CI: 0.84–1.44).

In summary, survival meta-analysis of RNA expression data from caBIG, GEO and TCGA repositories showed that patients with high expression of *MYO1E* had lower survival and increased risk of mortality for NSCLC (n = 1925) and LUAD (n = 719), but not for LUSC (n = 524).

### Survival analysis for DNA methylation and RNA expression (TCGA)

To study the association of MYO1E DNA methylation and survival using TCGA data, we split patients into high and low MYO1E DNA methylation groups using the median beta value of the cg13887966 probe.

**Figure 4:**
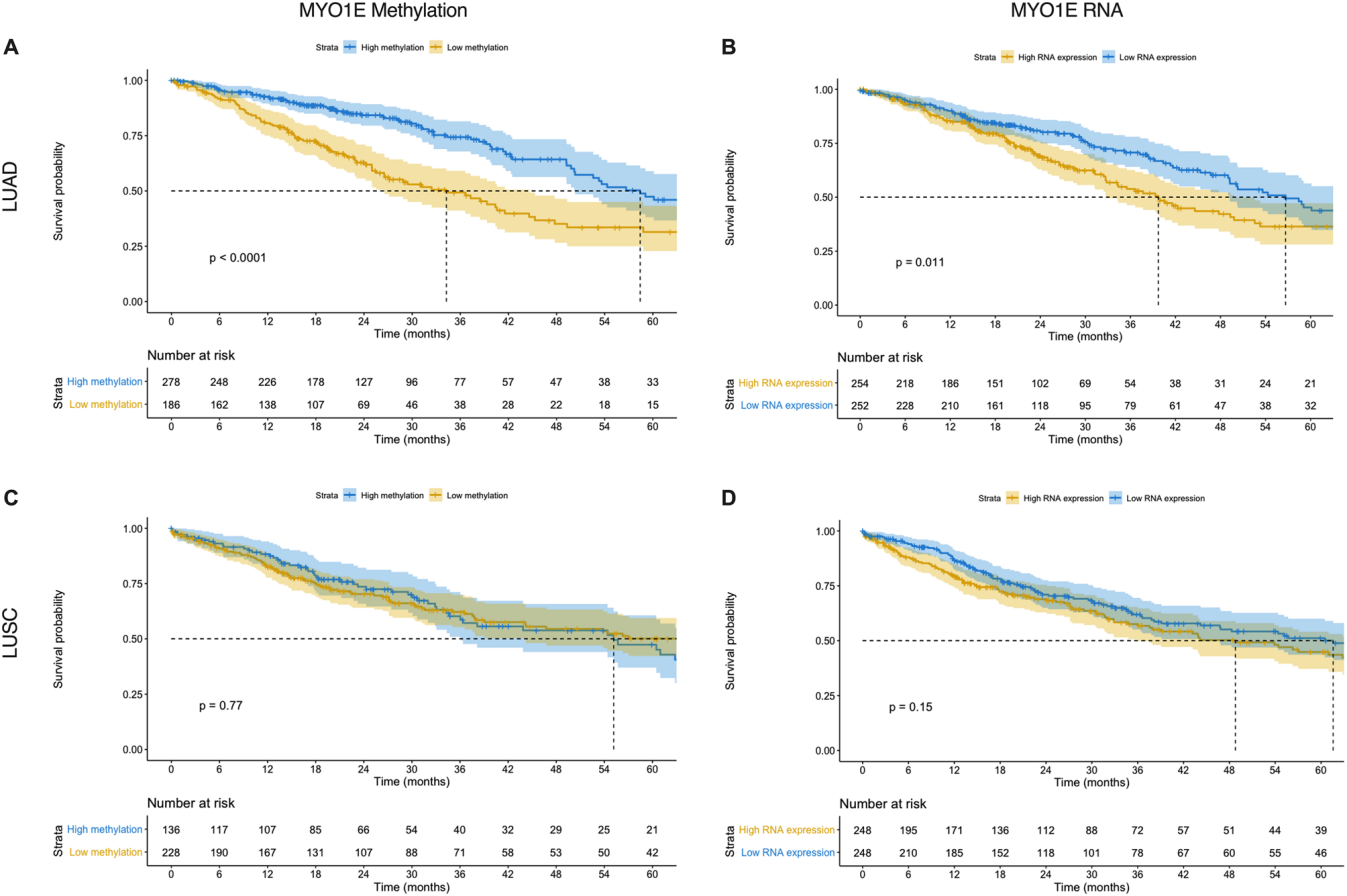
Patients with low DNA methylation and high RNA expression of *MYO1E* have significantly shorter median survival time in LUAD, but not in LUSC. Kaplan Meier survival curves with log-rank p values and numbers at risk when comparing high vs low MYO1E DNA methylation on the left and high vs low *MYO1E* RNA expression on the right for LUAD (top row) and LUSC (bottom row). (**A**) High vs low MYO1E DNA methylation for LUAD. (**B**) High vs low *MYO1E* RNA expression for LUAD. (**C**) High vs low MYO1E DNA methylation for LUSC. (**D**) High vs low *MYO1E* RNA expression for LUSC.

The median OS for the LUAD TCGA cohort was 49 months with 88% survival rate at one year, 75% at 2 years and 40% at five years. When comparing overall survival for patients with high vs low MYO1E DNA methylation in LUAD, log rank analysis showed statistically significant differences (p < 0.0001) with a median survival of 34 and 58 months for patients with low vs high MYO1E DNA methylation in LUAD, respectively (Fig. 4). The 1-, 2- and 5-year survival rates for the low MYO1E DNA methylation group were 81, 63, and 31% respectively compared to 93, 84, and 47% in the high methylation group. For patients with high vs low *MYO1E* RNA expression in LUAD, log rank analysis showed statistically significant differences (p = 0.011) with a median survival of 40 and 57 months for high and low *MYO1E* RNA expression, respectively (Fig. 4). The 1-, 2- and 5-year survival rates for the high *MYO1E* RNA expression in the LUAD group were 85, 69, and 36% respectively compared to 90, 81, and 45% for the low *MYO1E* RNA expression in LUAD group. Survival analysis of MYO1E DNA methylation and RNA expression in LUSC cohort did not show significant differences (Fig. 4).

In summary, TCGA data showed that low MYO1E methylation and high *MYO1E* RNA expression have shorter median survival times for LUAD, but not for LUSC.

### Survival analysis for DNA methylation and RNA expression (Validation cohort)

To validate our findings, we collected an independent cohort of 127 NSCLC patients from two medical centers (University of Illinois Chicago, USA and University Hospital of Patras, Greece). This validation cohort contained 66 LUAD and 55 LUSC samples. DNA methylation of MYO1E was measured by qMSP with primers designed near the Illumina DNA methylation probe cg13887966, and the RNA expression was measured by qPCR. Survival analysis on the independent validation dataset confirmed that patients with high *MYO1E* RNA expression have significantly shorter median survival time in LUAD, but not in LUSC and that low MYO1E DNA methylation has a trend towards shorter survival in LUAD but not in LUSC. (Supplemental Fig. 6). When comparing OS for patients with high vs low *MYO1E* RNA expression on the validation dataset in LUAD, log rank analysis showed statistically significant differences (p = 0.018) with a median survival of 34 and 58 months for LUAD patients with low vs high *MYO1E* RNA expression in LUAD, respectively (Supplemental Fig. 6). The 1-, 2- and 5-year survival rates for the low *MYO1E* RNA group were 87, 84, and 73% respectively compared to 88, 69, and 35% in the high *MYO1E* RNA group. We were able to confirm our observation made in TCGA that high *MYO1E* RNA expression was associated with poor survival in LUAD, but not in LUSC.

### Multivariate Cox analysis for DNA methylation and RNA expression (TCGA)

**Figure 5:**
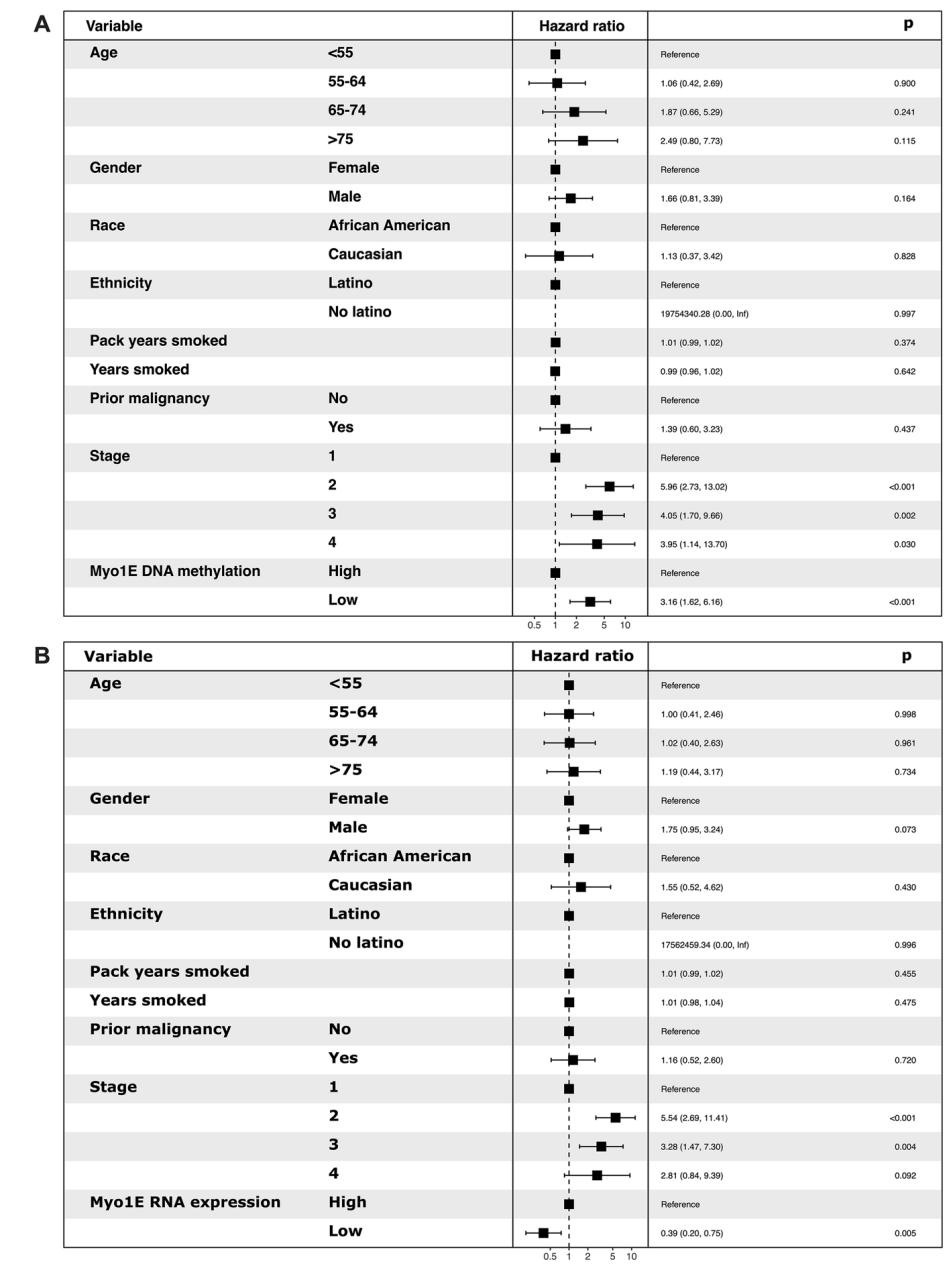
Low DNA methylation and high RNA expression of *MYO1E* are associated with increased mortality risk in LUAD. Forest plot for multivariate Cox proportional hazard analysis weighted for age, sex, race, Hispanic ethnicity, number of pack years smoked, number of years smoked, prior malignancy, histology, and stage in LUAD. (**A**) MYO1E DNA methylation, (**B**) *MYO1E* RNA expression.

Multivariate Cox proportional hazard analysis weighted for age, sex, race, Hispanic ethnicity, number of pack*years smoked, number of years smoked, prior malignancy, histology, and stage was applied to DNA methylation and expression of MYO1E in LUAD and LUSC on the TCGA data. In LUAD, low MYO1E DNA methylation was associated with increased mortality risk HR 3.16 (95% CI: 1.62–6.16) (p < 0.001) by the analysis weighted for age, sex, race, Hispanic ethnicity, number of pack*years smoked, number of years smoked, prior malignancy, histology, and stage (Fig. 5). On multivariate Cox analysis, low *MYO1E* RNA expression showed a significant association with decreased mortality risk in LUAD HR 0.39 (95% CI: 0.20–0.75) (p = 0.005) (Fig. 5). In LUSC, this analysis showed no significant association with survival and risk of mortality (Supplemental Fig. 7). In summary, TCGA data showed that low DNA methylation and high mRNA expression of *MYO1E* are associated with an increased risk of mortality for LUAD, but not for LUSC.

### Multivariate Cox analysis for DNA methylation and RNA expression (validation data)

Multivariate Cox proportional hazard analysis weighted for age, sex, race, number of pack*years smoked, and stage was applied to DNA methylation and expression of MYO1E in LUAD and LUSD on the validation data. This analysis confirmed our findings made in TCGA that high RNA expression of *MYO1E* was associated with increased mortality risk in LUAD and that low DNA methylation MYO1E showed a trend towards association with increased mortality risk in LUAD (Supplemental Fig. 8).

### Proof of concept of MYO1E as a potential liquid biopsy biomarker

Using the methodology described in previous publications from this lab^31,32^, MYO1E DNA methylation from 48 plasma samples showed a trend towards significant longer survival duration among those patients with high MYO1E DNA methylation with a 1-year survival rate of 96% vs 79%, 2-year survival rate of 96% vs 65% and a 5-year survival rate of 80% vs 51% among high vs low DNA methylation respectively (p = 0.075) (Supplemental Fig. 9). When looking into *MYO1E* RNA expression from plasma samples there were no statistically significant differences in survival time due to sample size. However, we can see that median survival time on high RNA expression was 33 months and that on low RNA expression median survival time was not reached, with a 1-year survival rate of 89% vs 80%, 2-year survival rate of 77% vs 72% and a 5-year survival rate of 77% vs 43% among low vs high RNA expression respectively (p = 0.25). These differences in survival time are not small, however sample size did not allow to reach statistical significance. A total sample size of 186 (with 93 high and 93 low MYO1E DNA methylation) will be needed to find significant association between methylation detection on MYO1E in plasma assuming a hazard ratio of 0.44, a power of 80%, a mortality probability within the study of 25% and a two-sided significance level of 5%.

## Discussion

The discovery of clinically useful biomarkers remains an unmet need in NSCLC. This study explored the clinical value of MYO1E DNA methylation and *MYO1E* RNA expression in NSCLC based on publicly available (TCGA) data, as well as data from two international medical centers (University of Illinois Chicago, USA and University Hospital of Patras, Greece). Our study showed the association of low DNA methylation as well as high RNA expression of *MYO1E* with a shorter median survival time and an increased risk of mortality for LUAD, but not for LUSC. To validate our findings, we additionally collected 127 NSCLC patient samples from two international centers. We measured DNA methylation and gene expression of *MYO1E,* and we were able to confirm the observation from TCGA data that *MYO1E* RNA expression was associated with shorter median survival time and an increased risk of mortality for LUAD. However, on the validation cohort, no statistically significant association was observed regarding the prognostic significance of MYO1E DNA methylation for both survival and mortality risk despite the trend being observed. This was mostly because of sample size since TCGA dataset had 515 LUAD patients, while the validation cohort included just 66 LUAD patients (Fig. 4 and Supplemental Fig. 5). The other possible explanations could be the difference in technologies that were used to measure DNA methylation and RNA expression for the TCGA dataset and the validation dataset. These results suggest *MYO1E* expression as a potential unfavorable epigenetic marker for LUAD. Additionally, the data suggested that MYO1E intronic DNA methylation regulates RNA expression. The regulation of gene expression by DNA methylation of intron CpG regions has been described in several studies across different tissues and species^33–36^. To the best of our knowledge, this is the first report that *MYO1E* expression and methylation is deregulated in NSCLC. While there is limited data on MYO1E methylation, few studies showed that hypomethylated MYO1E is associated with more aggressive forms of cancer. One study showed that decreased CpG site methylation and increased mRNA expression of *MYO1E* are associated with recurrence of hepatocellular carcinoma^37^, while hypomethylation of MYO1E has been reported in dermal fibroblasts in diffuse and limited cutaneous systemic sclerosis^38^*.*

*MYO1E* is a non-muscle class I myosin and an actin-dependent molecular motor that binds to the plasma membrane and serves as a scaffold for membrane protrusions contributing to membrane remodeling during endocytosis and exocytosis and cell migration and cell invasion^13–16^. Different studies have explored the importance of myosins and their potential role in cancer regulating tumor formation, cell invasion, migration and metastasis^17^. Non-muscle myosins are involved in drug resistance in melanoma by protecting tumor cells from reactive oxygen species and DNA damage and their expression was associated with cell survival regardless of the therapy^18,19^. Furthermore, in a breast cancer murine model, mice lacking MYO1E had tumors with increased differentiation and reduced proliferation^20^. Additionally, *MYO1E* expression has been shown to be correlated with poor prognosis in patients with invasive breast cancer^21^. Despite this association with aggressiveness, the role of *MYO1E* expression in NSCLC has not been previously explored. This study showed the utility of MYO1E DNA methylation detection in predicting NSCLC patients’ survival and suggested that changes in MYO1E methylation and expression in LUAD have an important role in the pathogenesis of this disease. Low *MYO1E RNA* expression in blood cells makes it an ideal candidate for a possible liquid biopsy marker. Such a marker has the potential to help with prognosis and monitoring in a non-invasive and more accessible manner.

Although the results of this study are very promising, we must acknowledge some limitations. A significant limitation is that data derived from TCGA or GTEx show an underrepresentation of minorities. Samples derived from African Americans account for less than 9% of total tumors in the TCGA data and less than 13% in the GTEx data. In addition, sample size of the validation cohort is another weak point of the current study. Furthermore, correlation studies cannot assume causality and therefore studies to prove the DNA methylation causes RNA expression silencing need to be performed to prove this inverse relationship. Lastly, validation studies using liquid biopsies are necessary to clarify whether MYO1E could be used as prognostic marker in liquid biopsies. Therefore, further studies are necessary to understand the mechanistic background of MYO1E in therapeutic resistance as well as microenvironment immune modulation.

This study suggests that changes in MYO1E methylation and expression in LUAD patients may have an essential role in lung cancer’s pathogenesis. It shows the utility of MYO1E DNA methylation and RNA expression in predicting survival for LUAD patients. Further studies are necessary to understand the role of *MYO1E* expression in chemotherapy resistance and microenvironment immune modulation.

## Methods

### Clinical data

Clinical data were obtained from The Cancer Genome Atlas (TCGA) for LUAD and LUSC^39,40^. Only primary tumor samples were queried. For external validation, we examined the clinical data and lung cancer tissues from two international centers, University of Illinois Chicago, USA and University Hospital of Patras, Greece. This study conforms with The Code of Ethics of the World Medical Association (Declaration of Helsinki). Institutional Review Board approval was obtained prior to study initiation (IRB #2017–1286 and #157/16.03.2017 for both centers respectively), and all patients signed informed consent. The inclusion criteria for the external validation included: (A) any adult with NSCLC of any stage, either biopsy proven or pathologically proven from surgical specimen from surgery involving a lobectomy, pneumonectomy, or greater resection. Stages will be defined according to revised TNM guidelines classification criteria^41^; (B) able to provide informed consent for this study. Exclusion criteria comprised: (A) patients who are pathologically diagnosed with small cell lung cancer, patients with metastatic disease by immunohistochemical criteria, as well as patients with other malignancies who preoperatively were incorrectly assumed to have primary NSCLC; (B) history of hereditary cancer; (C) radiotherapy or chemotherapy treatment had been given prior to surgical resection; (D) any adult lacking capacity to consent; (E) any patient < 30 years old; (F) pregnant patients. Collected biological variables included: age, sex, race, pack*year smoked, histology and TNM cancer stage. Baseline demographic characteristics of the groups were compared with the Wilcoxon rank sum test for continuous variables and Fisher's exact test for categorical variables. Statistically significant differences were defined for p-value < 0.05. The reporting of this study conforms to the Strengthening the Reporting of Observational Studies in Epidemiology statement^42^.

### Gene expression data

Gene expression data was obtained from The Cancer Genome Atlas (TCGA) Illumina HiSeq for lung adenocarcinoma (LUAD) and lung squamous cell carcinoma (LUSC) using the TCGAbiolinks R package^39,40^. Only primary solid tumor samples were queried. We performed an Array Array Intensity correlation (AAIC) with a threshold set to 0.6 to filter out samples based on Spearman’s correlation. mRNA transcripts were normalized using the EDASeq package based on gcContent^43^. Using the normalized mRNA transcripts, we dichotomized the samples into low and high *MYO1E* RNA expression groups based on the median *MYO1E* expression level. For external validation, RNA was extracted from four 5 μm slides of neoplastic FFPE tissue specimens from NSCLC patients using RNeasy FFPE kit (Qiagen, Cat No: 73504) following manufacturer’s instructions. RNA samples were then treated with DNase (Thermo Fisher scientific, Cat. No AM2222), quantified using a Nanodrop-1000 spectrophotometer (Thermo Fisher scientific, Cat. No ND-1000) and were stored at − 80 °C. A total of 2 μg of RNA was reverse transcribed into cDNA using High-Capacity cDNA Reverse Transcription Kit (Thermo Fisher Scientific, Cat. No.4368814). Each reverse transcription reaction contained 10μL of extracted cellular RNA, 2μL 10X RT buffer, 0.8 μL 25X dNTP mix, 2μL 10X Random Primers, 1μL MultiScribe Reverse Transcriptase and 4.2μL water making the total volume 20μL. The reverse transcription was performed on a SimpliAmp Thermal cycler (Thermo Fisher scientific, Cat. No A24811) at 25 °C for 10 min, 37 °C for 120 min, and 85 °C for 5 min. cDNA was diluted in DEPC water with a final volume of 100 μl and stored at −20 °C. Expression levels of *MYO1E* were quantified by real-time PCR (qPCR) assay. PowerUp SyBr Green Master Mix (Thermo Fisher Scientific, Cat. No. A25741) was used for the quantification along with specific primers designed by us (Supplemental Table 3). The qPCR reactions were carried out in triplicate in a total volume of 20 μl in QuantStudio 3 Systems (Thermo Fisher Scientific, Cat. No. A28567). A no template control was used in all reactions. The thermocycling conditions were as follows: 50 °C for 2 min 95 °C for 10 min, 40 cycles at 95 °C for 15 s and at 60 °C for 1 min. β-Actin was used as a reference gene. Primers for *MYO1E* and β-Actin were synthesized by Integrated DNA Technologies (IDT, Coralville, IA). The relative expression levels of *MYO1E* were calculated using the ΔΔCt method as described previously^31^.

### DNA methylation data

DNA methylation data generated via Illumina Human Methylation 450K array was obtained from TCGA for LUAD and LUSC using the TCGAbiolinks R package^39,40^. Only primary solid tumors were queried. 53 CpG sites were found for the MYO1E gene. Of these probes, cg13887966 showed the highest correlation between DNA methylation and RNA expression using Spearman’s rank correlation coefficient. Methylation data were dichotomized into high and low MYO1E DNA methylation groups based on the median methylation at the cg13887966 probe. For the external validation, we performed methylation on beads followed by qMSP of the cg13887966 probe using the same methodology as previously described^31,32^ (Supplemental Table 4).

### Functional relationship between DNA methylation and RNA expression

We queried the Gene Expression Omnibus (GEO) repository for studies using human cell lines assessing the effect of 5-Azacitidine on MYO1E DNA methylation and RNA expression. For DNA methylation we used cell lines from GEO accession GSE202559^23^ and for RNA expression we used cell lines from GEO accession GSE62955 and GSE62958^24^.

### Differential expression analysis

After filtering the normalized mRNA transcripts with a 0.25 quantile method, we performed a differential expression analysis on the low and high *MYO1E* RNA samples based on median expression levels using the TCGAbiolinks R package^39,40^. The results were visualized in a volcano plot with an FDR cutoff of 10^−5^ and log_2_FC of 3.

### Correlation expression analysis

Using the TCGA RNA seq data from LUAD and LUSC (Illumina HiSeq, RSEM normalized counts) we used Spearman’s rank correlation coefficient to assess the RNA expression correlation between *MYO1E* and some of the most relevant oncogenic pathway genes in lung cancer including: *MAP2K1, MAPK1, MAPK3, KRAS, mTOR, AKT1, AKT2, RPS6, CDKN2A*, *RB1, p53, MYC, STK11, EGFR and KEAP1*^25,26^.

### Normal tissue RNA expression

The RNA sequencing data used was obtained from the Genotype-Tissue Expression (GTEx) Project Portal dbGaP accession phs000424.v8.p2^29,44,45^. The extensive collection of RNA sequences from the GTEx project represents more than 10,000 samples from 838 healthy individuals, spanning across more than 50 different normal primary tissues.

### Survival analysis

For survival meta-analysis of NSCLC, we used Kaplan–Meier Plotter (www.kmplot.com/lung), an online tool developed by Gyorffy et al.^30^ to analyze the effect of *MYO1E* RNA expression in survival. This tool performs a survival meta-analysis of data from the cancer Biomedical Informatics Grid (caBIG), Gene Expression Omnibus (GEO), and The Cancer Genome Atlas (TCGA) repositories combined (n = 1925). For *MYO1E* gene expression, this tool uses the Affymetrix probe 203072_at.

We also performed survival analysis for the TCGA data and the external validation data. Kaplan–Meier curves were used to estimate overall survival between high and low *MYO1E* RNA expression and high and low MYO1E DNA methylation groups. Survival differences were compared using the two-tailed log-rank test for significance. As aforementioned, using the normalized mRNA transcripts, we dichotomized the samples into high and low *MYO1E* RNA expression groups based on the median *MYO1E* expression level. Methylation data were dichotomized into high and low MYO1E DNA methylation groups based on the median methylation at the cg13887966 probe. Statistically significant differences were defined for p-value < 0.05. Association with survival was quantified using hazard ratios (HRs) with 95% confidence intervals (CIs) assessed with univariate and multivariate Cox proportional hazard models. R statistical software, version 4.0.0, Vienna, Austria was used for the analysis^46^.

### Ethical approval and consent to participate

This study conforms with The Code of Ethics of the World Medical Association (Declaration of Helsinki). Institutional Review Board approval was obtained prior to study initiation (IRB #2017-1286 and #157/16.03.2017 for both study centers respectively), and all patients signed informed consent.

## Supplementary Information

Supplementary Table 1.

Supplementary Table 2.

Supplementary Table 3.

Supplementary Table 4.

Supplementary Figures.

## Data Availability

The dataset supporting the conclusions of this article is available in the TCGA repository: https://cancergenome.nih.gov/. The RNA sequencing data used was obtained from the Genotype-Tissue Expression (GTEx) Project Portal dbGaP accession phs000424.v8.p2. Functional cell lines studies were obtained from Gene Expression Omnibus (GEO) accession numbers GSE202559 GSE62955 and GSE62958.

## Abbreviations

AKT: AKT serine/threonine kinase
ALK: Anaplastic lymphoma kinase
caBIG: Cancer biomedical informatics grid
CDKN2A: Cyclin-dependent kinase inhibitor 2A
CHGA: Chromogranin A
CI: Confidence interval
EGFR: Epidermal growth factor receptor
FFPE: Formalin-fixed paraffin-embedded
GEO: Gene expression omnibus
GTEx: Genotype-tissue expression
HR: Hazard ratio
KRAS: Kirsten rat sarcoma
LUAD: Lung adenocarcinoma;
LUSC: Lung squamous cell carcinoma
MAP2K1: Mitogen-activated protein kinase kinase 1
MAPK: Mitogen-activated protein kinase
mTOR: Mammalian target of rapamycin
MYO1E: Myosin 1E
NPY: Neuropeptide Y
NSCLC: Non-small cell lung carcinoma
OS: Overall survival
qPCR: Quantified by real-time polymerase chain reaction
RB1: Retinoblastoma 1
RPS6: Ribosomal protein S6
TCGA: The Cancer Genome Atlas
ΔΔCt: Delta delta cycle threshold
