# Supplementary material for: Myo1e overexpression in lung adenocarcinoma is associated with increased risk of mortality
Source: Sci Rep. 2023 Mar 13;13:4107. doi: 10.1038/s41598-023-30765-y (PMC10011530; doi:10.1038/s41598-023-30765-y)
Supplement: Supplementary file 1 — Supplementary Table 1. [file 41598_2023_30765_MOESM1_ESM.docx]

### Supplemental Table 1. Baseline Characteristics of the 1017 NSCLC subjects

|  | **Patient Characteristics** | **High Myo1E RNA**  **(N=509)** | **Low Myo1E RNA**  **(N=508)** | ***p* Value** |
| --- | --- | --- | --- | --- |
|  | Age at diagnosis (years) (IQR) | 68 (60-74) | 67 (60-73) | 0.513 |
|  | Sex |  |  |  |
|  | Male (%) | 302 (59%) | 307 (60%) | 0.749 |
|  | Female (%) | 207 (41%) | 201 (40%) |  |
|  | Race |  |  |  |
|  | Caucasians (%) | 368 (72%) | 370 (73%) | 0.981 |
|  | African Americans (%) | 40 (8%) | 42 (8%) |  |
|  | Asians (%) | 9 (2%) | 8 (2%) |  |
|  | American Indian or Alaska Native (%) | 1 (<1%) | 0 (0%) |  |
|  | Non reported (%) | 91 (8%) | 88 (17%) |  |
|  | Ethnicity: Hispanic or Latino (%) | 12 (2%) | 3 (1%) | 0.07 |
|  | Pack-year (IQR) | 40 (28-60) | 44 (30-60) | 0.289 |
|  | Years smoked (IQR) | 39 (26-45) | 37 (28-45) | 0.887 |
|  | Prior malignancy | 81 (16%) | 62 (12%) | 0.104 |
|  | Histology |  |  |  |
|  | Adenocarcinoma (LUAD) (%) | 258 (51%) | 257 (51%) | 1 |
|  | Squamous-cell (LUSC) (%) | 251 (49%) | 251 (49%) |  |
|  | Stage |  |  |  |
|  | I (%) | 241 (48%) | 279 (56%) | 0.036 |
|  | II (%) | 162 (32%) | 122 (24%) |  |
|  | III (%) | 86 (17%) | 82 (16%) |  |
|  | IV (%) | 17 (3%) | 16 (3%) |  |
|  |  |  |  |  |

*Abbreviations*: Interquartile range: IQR.
