# Supplementary material for: Myo1e overexpression in lung adenocarcinoma is associated with increased risk of mortality
Source: Sci Rep. 2023 Mar 13;13:4107. doi: 10.1038/s41598-023-30765-y (PMC10011530; doi:10.1038/s41598-023-30765-y)
Supplement: Supplementary file 3 — Supplementary Table 3. [file 41598_2023_30765_MOESM3_ESM.docx]

### Supplemental Table 3. RNA Expression Primers sequences.

| **Gene** | **Forward 5' - 3'** | **Reverse 5' - 3'** | **Size (bp)** | **Temperature** |
| --- | --- | --- | --- | --- |
| MYO1E | TGGATTTCCTCAAGGTCCCG | GGCCGACTGGTTGTTTGTCT | 64 | 61 |
| B-ACTIN | GAGCACAGAGCCTCGCCTTT | TCATCATCCATGGTGAGCTGG | 70 | 61 |
