# Supplementary material for: Myo1e overexpression in lung adenocarcinoma is associated with increased risk of mortality
Source: Sci Rep. 2023 Mar 13;13:4107. doi: 10.1038/s41598-023-30765-y (PMC10011530; doi:10.1038/s41598-023-30765-y)
Supplement: Supplementary file 5 — Supplementary Figures. [file 41598_2023_30765_MOESM5_ESM.docx]

### Supplemental Figure 1: 5-AZA significantly decreases MYO1E DNA methylation and increases MYO1E RNA expression in human cell lines.


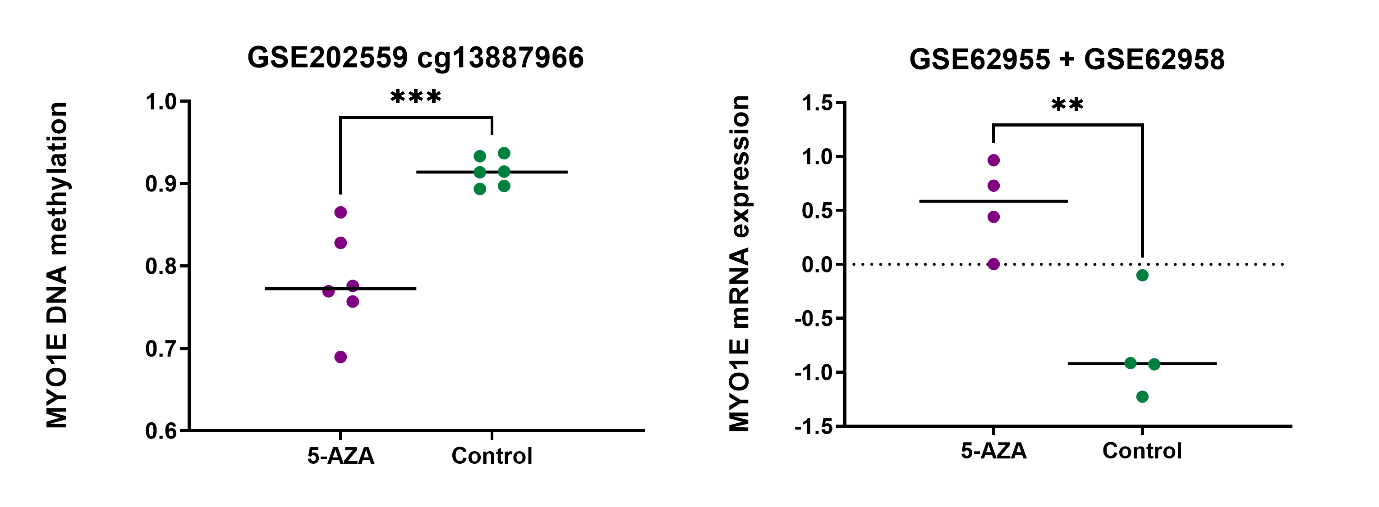


### Supplemental Figure 2. *MYO1E* RNA expression was directly correlated with the RNA expression of MAPK/ERK, MTOR and EGFR pathways and inversely correlated with RPS6 pathway on both LUAD and LUSC. Heatmap showing Spearman’s correlation coefficients for *MYO1E* RNA expression with some of the most relevant oncogenic pathway genes in lung cancer. Red represents direct correlation and green represents inverse correlation (p<0.1 “.”, p<0.05 “*”; p<0.01 “**”; p<0.001 “***”).


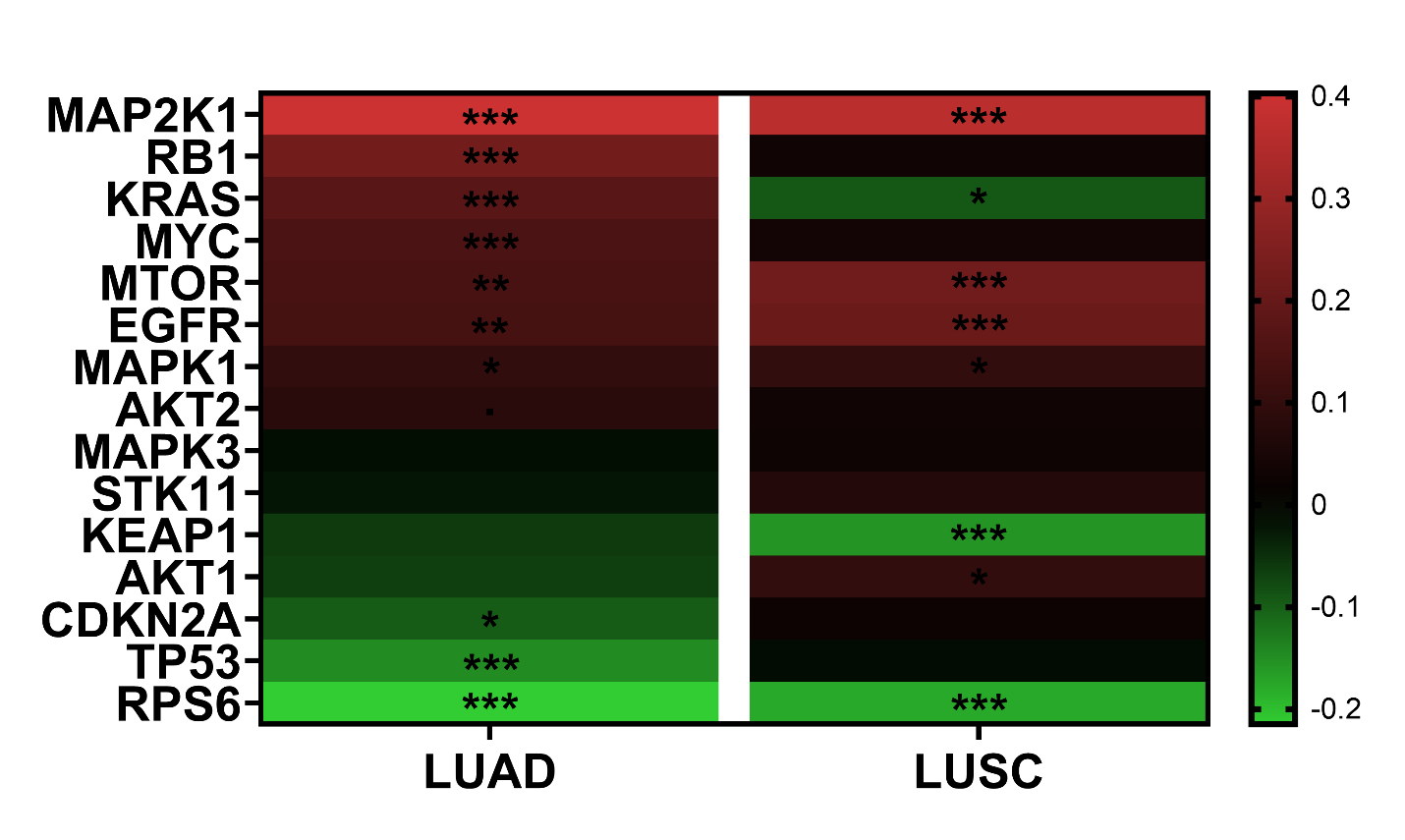


### Supplemental Figure 3. Violin plots showing *MYO1E* RNA expression values in different normal human tissues from the GTEx project shown in logarithmic scale of TPM (Transcripts Per Million). Boxplots are shown as median and 25^th^ and 75^th^ percentiles.


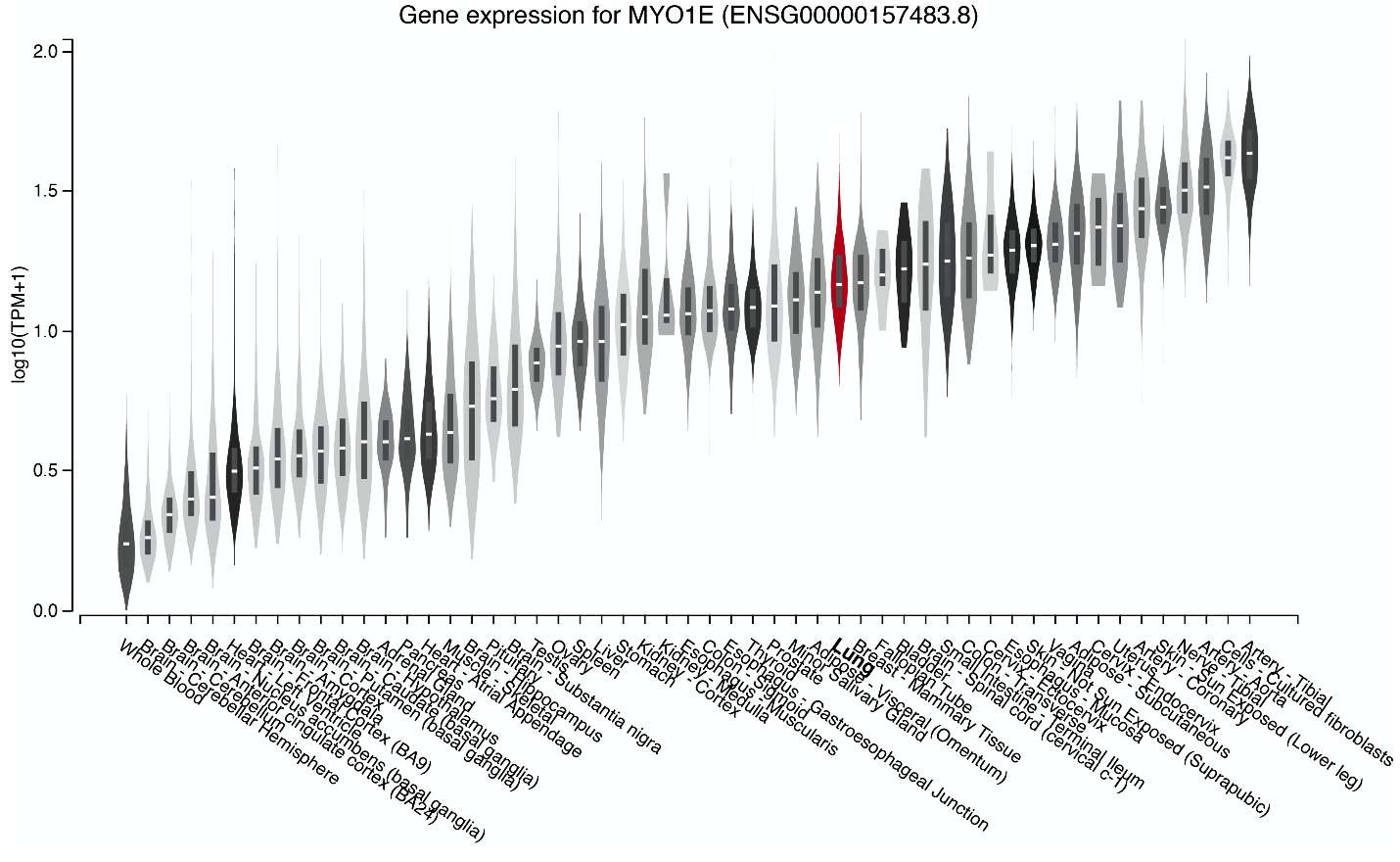


### Supplemental Figure 4. High *MYO1E* RNA expression has shorter median survival time for NSCLC and LUAD but not LUSC. Kaplan Meier survival curves from Kaplan-Meier Plotter's meta-analysis data ([www.kmplot.com/lung](http://www.kmplot.com/lung)) for NSCLC, LUAD and LUSC comparing high vs low *MYO1E* RNA expression with univariate and multivariate Cox proportional hazard analysis weighted adjusting for histology, stage, sex and smoking history.


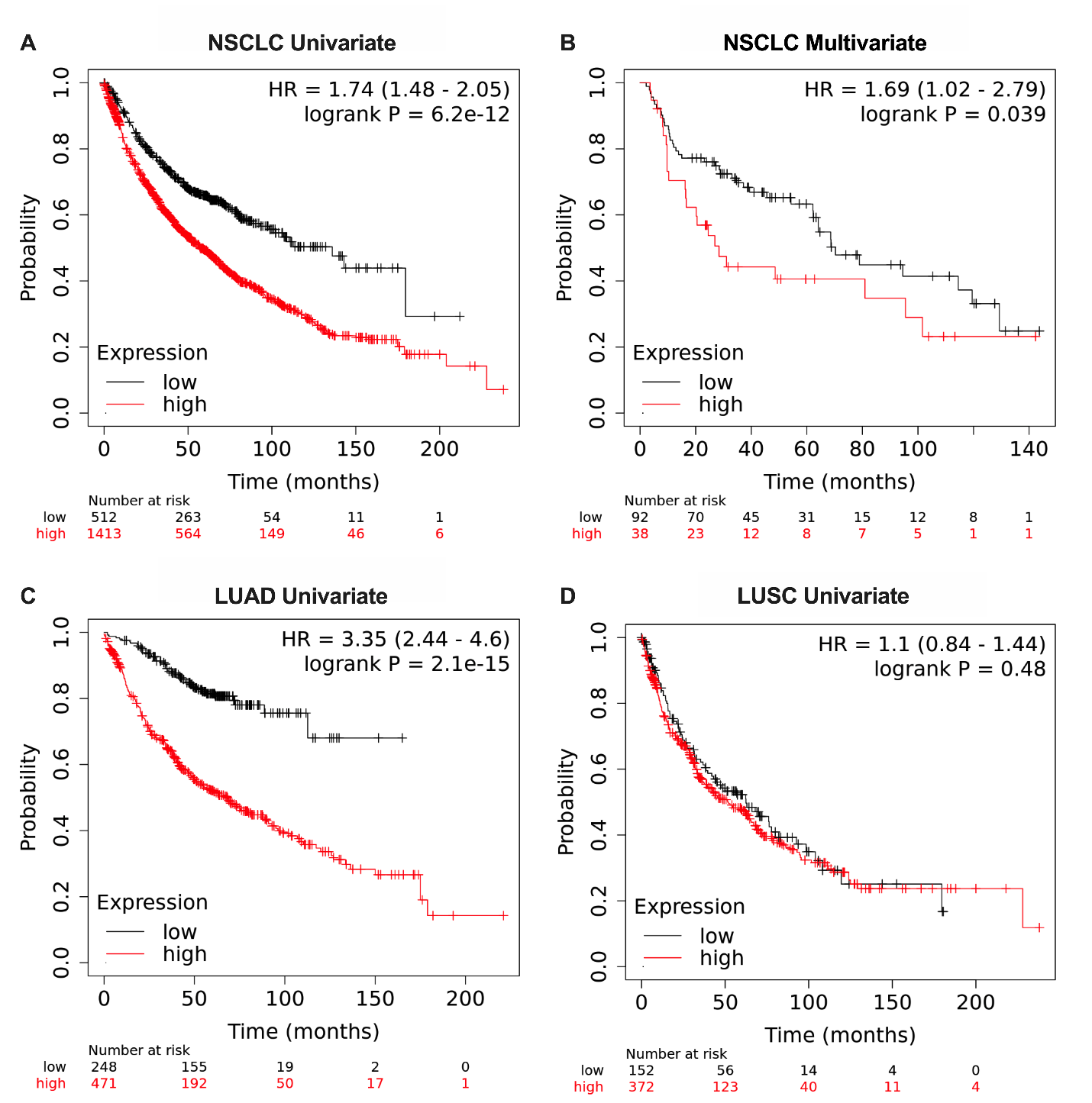


### Supplemental Figure 5. Patients with high *MYO1E* RNA expression have a shorter median survival time for LUAD and it is associated with an increased risk of mortality in LUAD. Kaplan Meier survival curves from meta-analysis data from caBIG, GEO and TCGA for LUAD comparing high vs low *MYO1E* RNA expression using multivariate Cox proportional hazard analysis weighted for histology, stage, sex and smoking history.


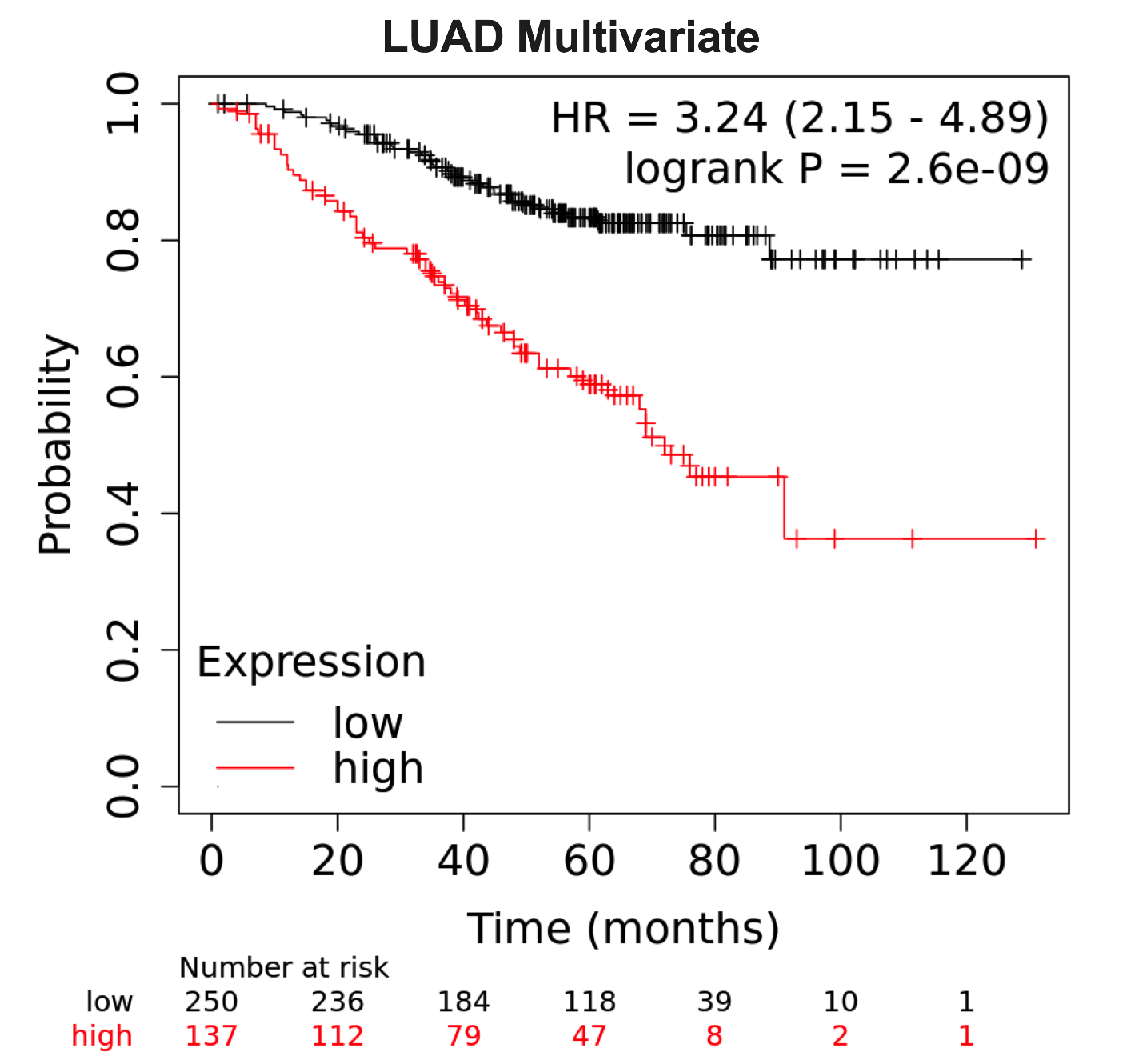


### Supplemental Figure 6. External validation data confirming that high RNA expression of *MYO1E* have significantly shorter median survival time in LUAD, but not in LUSC and that low DNA methylation has a trend towards shorter survival. Kaplan Meier survival curves with log-rank p values and numbers at risk when comparing high vs low MYO1E DNA methylation on the left and high vs low *MYO1E* RNA expression on the right for LUAD (top row) and LUSC (bottom row).


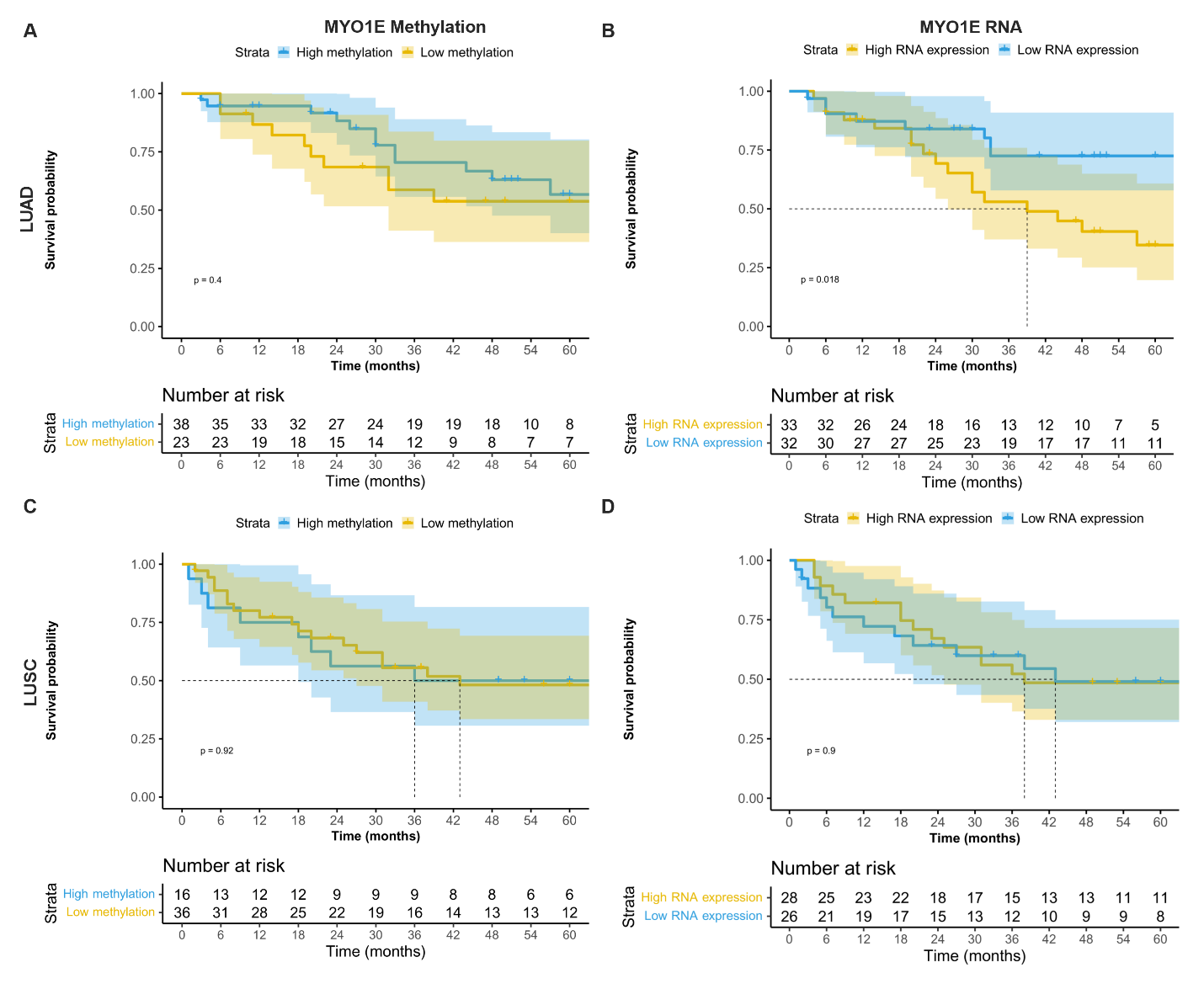


### Supplemental Figure 7. MYO1E DNA methylation and *MYO1E* RNA expression are not associated with mortality risk in LUSC. Forest plot for multivariate Cox proportional hazard analysis weighted for age, sex, race, Hispanic ethnicity, number of pack years smoked, number of years smoked, prior malignancy, histology, and stage in LUSC.


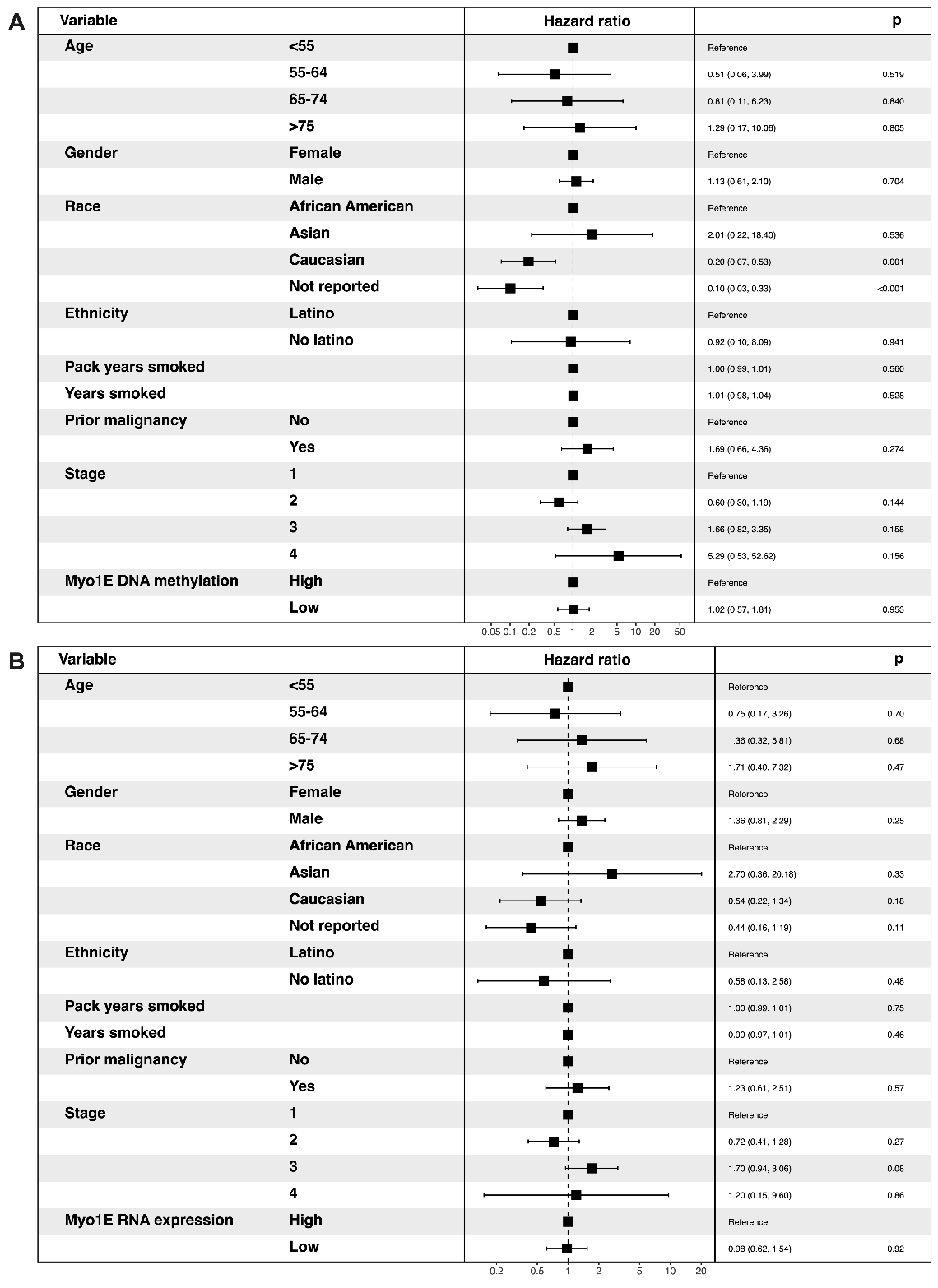


### Supplemental Figure 8. External validation data showing that high RNA expression of *MYO1E* is associated with increased mortality risk in LUAD and that low DNA methylation MYO1E shows a trend towards association with increased mortality risk in LUAD. Forest plot for multivariate Cox proportional hazard analysis weighted for age, sex, race, Hispanic ethnicity, number of pack years smoked, number of years smoked, prior malignancy, histology, and stage in LUAD.


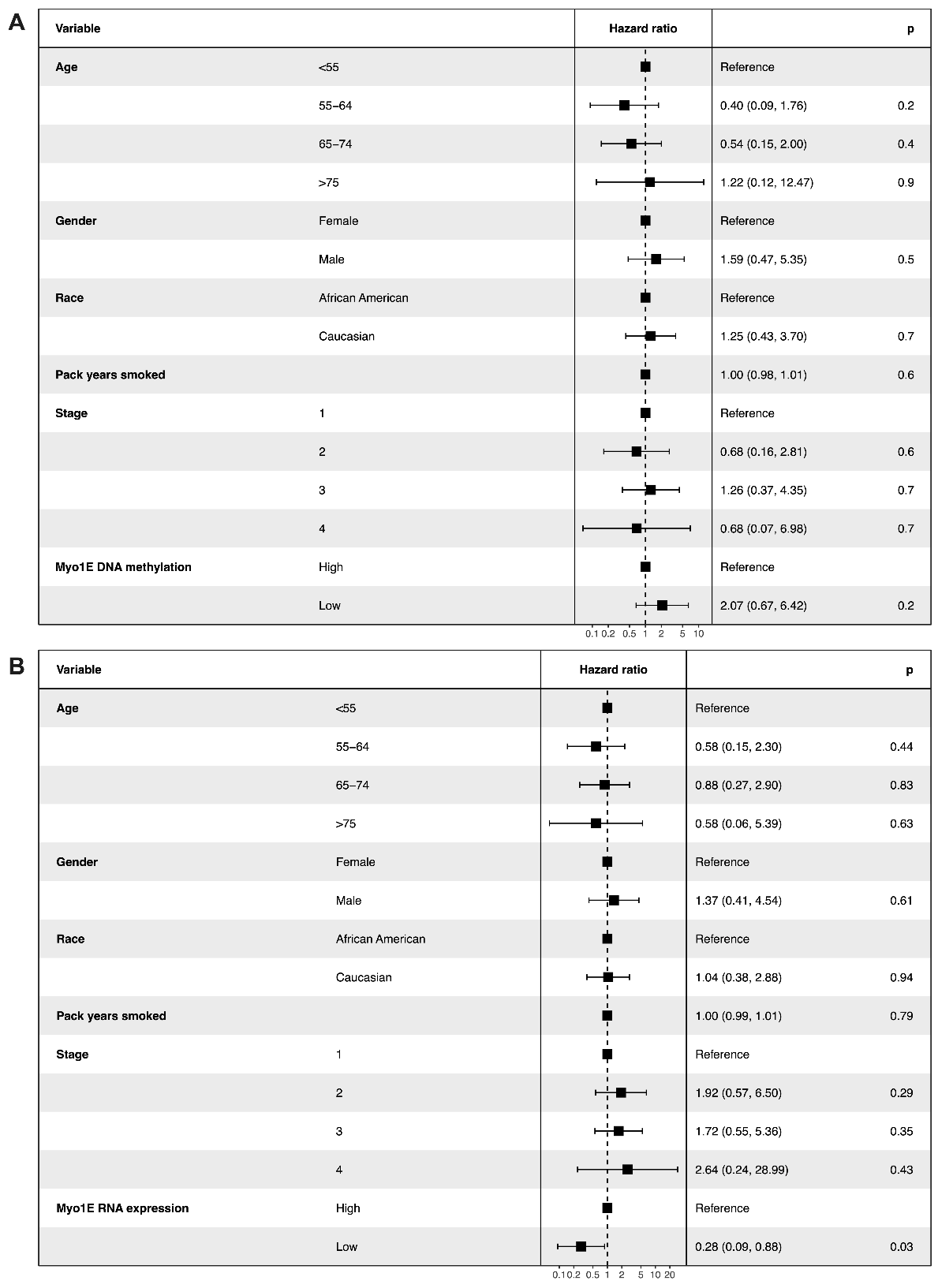


### Supplemental figure 9. Proof of concept of the potential of MYO1E DNA methylation and RNA expression to be used as circulating tumor marker in liquid biopsies. Kaplan Meier and univariate regression analysis from plasma samples showing **A**. Trend towards shorter survival duration for low MYO1E DNA methylation (p=0.075) **B**. Non-statistically significant association between MYO1E DNA methylation and mortality risk (HR 0.44, 95% CI: 0.12-1.66). **C**. Non-statistically significant survival duration differences for low vs high *MYO1E* RNA expression and **D**. Non-statistically significant association between *MYO1E* RNA expression and mortality risk (HR 2.0, 95% CI: 0.60-6.69).
